# Supplementary material for: The Small RNA Universe of Capitella teleta
Source: Front Mol Biosci. 2022 Feb 25;9:802814. doi: 10.3389/fmolb.2022.802814 (PMC8915122; doi:10.3389/fmolb.2022.802814)
Supplement: Supplementary file 1 [file DataSheet1.ZIP › Supplement/known/CAPTEscaffold_794_29004.pdf]

Provisional ID	:	CAPTEscaffold_794_29004
Score total	:	2509.3
Score for star read(s)	:	3.9
Score for read counts	:	2499.3
Score for mfe	:	1.5
Score for randfold	:	1.6
Score for cons. seed	:	3
Total read count	:	4914
Mature read count	:	4804
Loop read count	:	15
Star read count	:	95

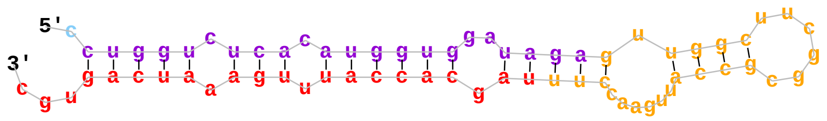

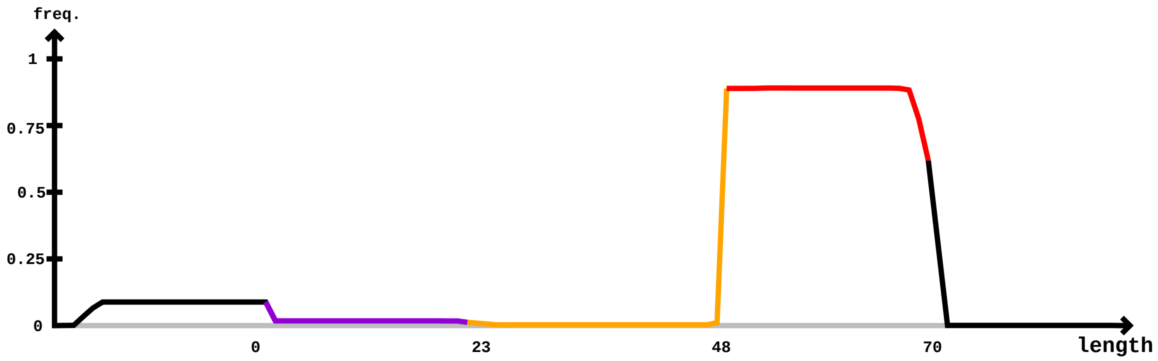


```
                              Star                                         Mature
5'- ugcaauauggaggcucggcgccuggucucacaugguggauagaguuggcuucggcgccauugaaccuuuagcaccauuugaaaucagugcuucuggcucuccuccgccuu  -3'   obs
    ugcaauauggaggcucggcgccuggucucacaugguggauagaguuggcuucggcgccauugaaccuuuagcaccauuugaaaucagugcuucuggcucuccuccgccuu         exp
    ugcaauauggaggcucggcgccuggucucacaugguggauagaguuggcuucggcgccauugaaccuuuagcaccauuugaaaucagugcuucuggcucuccuccgccuu         known
    .((.....(((((((.((.(((((((.(((.((((((..(((((.((((......)))).......))))).)))))).))).))))).)).)).))).))))...))...    reads      mm        sample

    ugcaauauggaggcucggcgc.........................................................................................    5          0         seq
    .gcaauauggagCcucggcgc.........................................................................................    1          1         seq
    .gcaauauggaggcuUggcgc.........................................................................................    1          1         seq
    .gcaauauggaggcucggcgc.........................................................................................    155        0         seq
    .gcaauauggaggcucAgcgc.........................................................................................    22         1         seq
    ..Uaauauggaggcucggcgc.........................................................................................    1          1         seq
    ..caauauggaggcucggcgc.........................................................................................    169        0         seq
    ..caauauAgaggcucggcgc.........................................................................................    1          1         seq
    ...aauauggaggcCcggcgc.........................................................................................    1          1         seq
    ...aauauggaggcucggcgc.........................................................................................    120        0         seq
    .....................cuggucucacauggugga.......................................................................    2          0         seq
    .....................cuggucucacaugguggaua.....................................................................    27         0         seq
    .....................cuggucucacaugguggauag....................................................................    1          0         seq
    .....................cuggucucacaugguggauaA....................................................................    2          1         seq
    .....................cuggucucacaugguggauaga...................................................................    62         0         seq
    .....................cuggucucacaugguggauagaUu.................................................................    1          1         seq
    ...........................................guuggcuucggcgccauugaaccuu..........................................    13         0         seq
    ............................................uuggcuucggcgccauugaaccu...........................................    2          0         seq
    ..............................................ggcuucggcgccauugaaccuuuagcacc...................................    1          0         seq
    ...................................................................uuagcaccauuugaaaucag.......................    4          0         seq
    ...................................................................uuagcaccauuugaaaucagu......................    6          0         seq
    ...................................................................uuagcaccauuugaaaucaguU.....................    2          1         seq
    ...................................................................uuagcaccauuugaaaucagug.....................    23         0         seq
    ...................................................................uuagcaccauuugaaaucagugc....................    10         0         seq
    ....................................................................uagcaccauuugaaauca........................    6          0         seq
    ....................................................................uagcaccauuugaaaucag.......................    29         0         seq
    ....................................................................Cagcaccauuugaaaucagu......................    1          1         seq
    ....................................................................Aagcaccauuugaaaucagu......................    1          1         seq
    ....................................................................uagcaccauuugaaaucagu......................    568        0         seq
    ....................................................................uagUaccauuugaaaucagu......................    1          1         seq
    ....................................................................uagcaccauuugaaaucagA......................    3          1         seq
```

```
                    Star                                              Mature
ugcaauauggaggcucggcgccuggucucacaugguggauagaguuggcuucggcgccauugaaccuuuagcaccauuugaaaucagugcuucuggcucuccuccgccuu
....................................................................uagcaccauuuAaaaucagu......................    1      1      seq
....................................................................uagcacUauuugaaaucagu......................    1      1      seq
....................................................................uagcaccauuAgaaaucagug.....................    1      1      seq
....................................................................uagcacUauuugaaaucagug.....................    2      1      seq
....................................................................uagcaccauuugaaaucaguA.....................    2      1      seq
....................................................................uagcaccauuuAaaaucagug.....................    2      1      seq
....................................................................uaUcaccauuugaaaucagug.....................    1      1      seq
....................................................................uagcacAauuugaaaucagug.....................    1      1      seq
....................................................................uagcaccauuugaaaucagug.....................    809    0      seq
....................................................................Aagcaccauuugaaaucagug.....................    3      1      seq
....................................................................uagcaccauuugaaaucaguUc....................    4      1      seq
....................................................................uagcaUcauuugaaaucagugc....................    2      1      seq
....................................................................uagcaccauuugaGaucagugc....................    1      1      seq
....................................................................uGgcaccauuugaaaucagugc....................    1      1      seq
....................................................................uagcaccauuugUaaucagugc....................    1      1      seq
....................................................................Gagcaccauuugaaaucagugc....................    2      1      seq
....................................................................uagcaccauuugaaaucagugG....................    16     1      seq
....................................................................uagcaccauuAgaaaucagugc....................    4      1      seq
....................................................................uagAaccauuugaaaucagugc....................    1      1      seq
....................................................................uagcacAauuugaaaucagugc....................    3      1      seq
....................................................................uagcaccauuuCaaaucagugc....................    1      1      seq
....................................................................uagcaccauuugaaaucagugU....................    17     1      seq
....................................................................uagcaccauuugaaauUagugc....................    3      1      seq
....................................................................uagcaccGuuugaaaucagugc....................    1      1      seq
....................................................................uagcaccauCugaaaucagugc....................    1      1      seq
....................................................................Cagcaccauuugaaaucagugc....................    1      1      seq
....................................................................uagcaccauuugaaaucagugc....................    3176   0      seq
....................................................................Aagcaccauuugaaaucagugc....................    17     1      seq
....................................................................uagcaccaCuugaaaucagugc....................    2      1      seq
....................................................................uagcGccauuugaaaucagugc....................    1      1      seq
....................................................................uagcacUauuugaaaucagugc....................    1      1      seq
....................................................................uagcaccauAugaaaucagugc....................    5      1      seq
....................................................................uagcaccauuugaaaucGgugc....................    1      1      seq
....................................................................uagcaccauuugaaauAagugc....................    2      1      seq
....................................................................uagcaccauuugGaaucagugc....................    3      1      seq
....................................................................uagcaccauuugCaaucagugc....................    1      1      seq
....................................................................uagcaccauuuAaaaucagugc....................    12     1      seq
....................................................................uagcaccauuugaaaucagugA....................    11     1      seq
....................................................................uagcaAcauuugaaaucagugc....................    1      1      seq
....................................................................uagcaccauuugaaaucagugcA...................    26     1      seq
....................................................................uagcaccauuugaaaucagugcu...................    1      0      seq
.......................................................................caccauuugaaaucagugc....................    1      0      seq
........................................................................accauuugaaaucagugc....................    6      0      seq
.........................................................................ccauuugaaaucagugcA...................    1      1      seq
..........................................................................................uucuggcucuccuccgccu.    2      0      seq
..........................................................................................uucuggcucuccuccgccA.    1      1      seq
```
